# Virtual reality for management of pain in hospitalized patients: A randomized comparative effectiveness trial

**DOI:** 10.1371/journal.pone.0219115

**Published:** 2019-08-14

**Authors:** Brennan Spiegel, Garth Fuller, Mayra Lopez, Taylor Dupuy, Benjamin Noah, Amber Howard, Michael Albert, Vartan Tashjian, Richard Lam, Joseph Ahn, Francis Dailey, Bradley T. Rosen, Mark Vrahas, Milton Little, John Garlich, Eldin Dzubur, Waguih IsHak, Itai Danovitch

**Affiliations:** 1 Cedars-Sinai Health System, Division of Health Services Research, Department of Medicine, Los Angeles, CA, United States of America; 2 Cedars-Sinai Graduate Program, Division of Health Delivery Science, Los Angeles, CA, United States of America; 3 Inpatient Specialty Program, Cedars-Sinai Health System, Los Angeles, CA, United States of America; 4 Department of Orthopaedics, Cedars-Sinai Health System, Los Angeles, CA, United States of America; 5 Department of Psychiatry, Cedars-Sinai Health System, Los Angeles, CA, United States of America; University of California San Diego, UNITED STATES

## Abstract

**Objectives:**

Therapeutic virtual reality (VR) has emerged as an effective, drug-free tool for pain management, but there is a lack of randomized, controlled data evaluating its effectiveness in hospitalized patients. We sought to measure the impact of on-demand VR versus “health and wellness” television programming for pain in hospitalized patients.

**Methods:**

We performed a prospective, randomized, comparative effectiveness trial in hospitalized patients with an average pain score of ≥3 out of 10 points. Patients in the experimental group received a library of 21 VR experiences administered using the Samsung Gear Oculus headset; control patients viewed specialized television programming to promote health and wellness. Clinical staff followed usual care; study interventions were not protocolized. The primary outcome was patient-reported pain using a numeric rating scale, as recorded by nursing staff during usual care. Pre- and post-intervention pain scores were compared immediately after initial treatment and after 48- and 72-hours.

**Results:**

There were 120 subjects (61 VR; 59 control). The mean within-subject difference in immediate pre- and post-intervention pain scores was larger in the VR group (-1.72 points; SD 3.56) than in the control group (-0.46 points; SD 3.01); this difference was significant in favor of VR (P < .04). When limited to the subgroup of patients with severe baseline pain (≥7 points), the effect of VR was more pronounced vs. control (-3.04, SD 3.75 vs. -0.93, SD 2.16 points; P = .02). In regression analyses adjusting for pre-intervention pain, time, age, gender, and type of pain, VR yielded a .59 (P = .03) and .56 (P = .04) point incremental reduction in pain versus control during the 48- and 72-hour post-intervention periods, respectively.

**Conclusions:**

VR significantly reduces pain versus an active control condition in hospitalized patients. VR is most effective for severe pain. Future trials should evaluate standardized order sets that interpose VR as an early non-drug option for analgesia.

## Introduction

Effective and safe pain management is an important challenge in the acute-care setting. Nearly half of hospitalized patients experience pain, of which one quarter is considered “unbearable.”[1] Pain treatment is traditionally based on pharmacological management, including opioids, which can yield inconsistent and sub-optimal results.[2] Data from the United States Center for Disease Control reveals that even one day of opioid therapy predicts a six percent risk of dependency one year later.[3] Thus, there is a pressing need for safe, effective, drug-free solutions for pain management in hospitalized patients.

**Fig 1:**
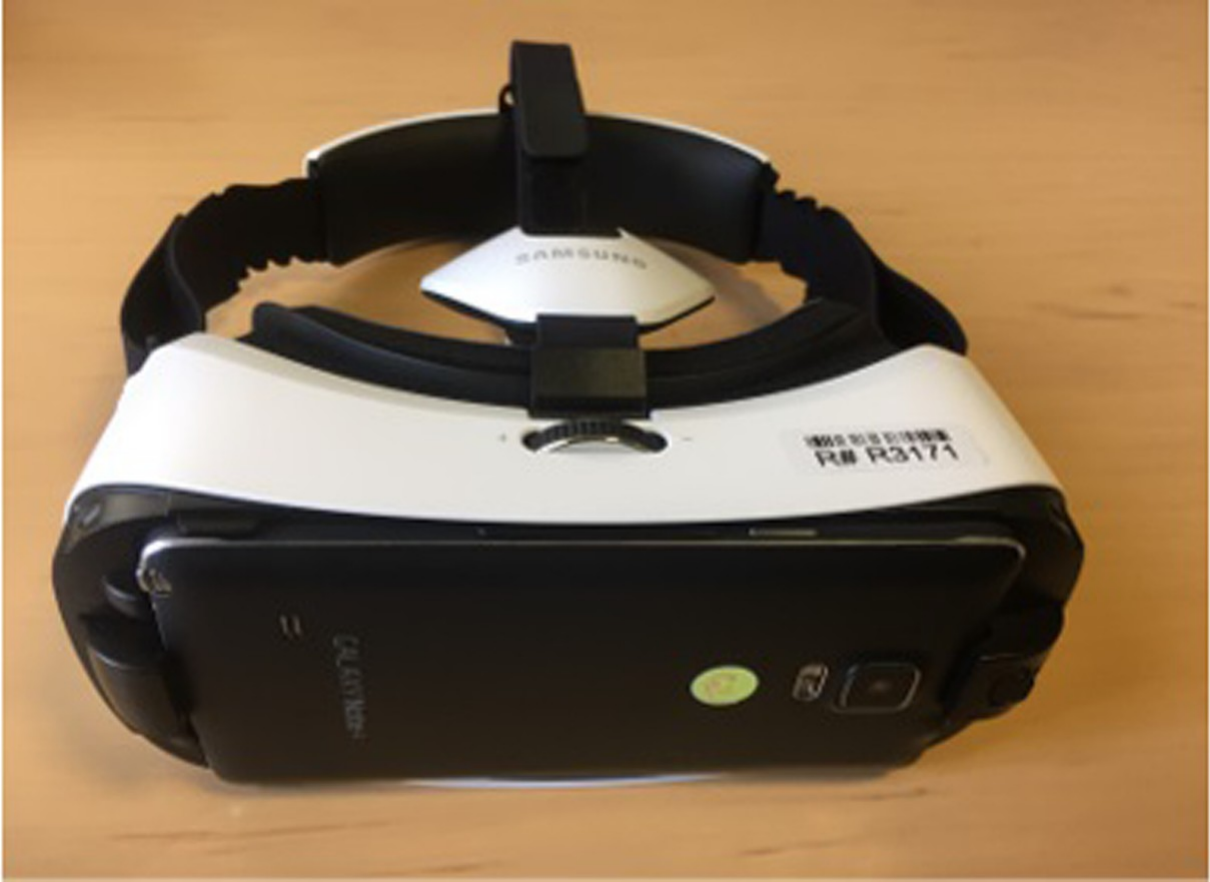
Samsung gear virtual realty headset.

Therapeutic virtual reality (VR) has emerged as an effective, non-pharmacological treatment modality for pain.[4, 5] Users of VR wear a head mounted display with a close-proximity screen that creates a sensation of being transported into lifelike, three-dimensional worlds (**Fig 1**). A proposed mechanistic theory of VR suggests that by stimulating the visual cortex while engaging other senses, VR acts as a distraction to limit the user’s processing of nociceptive stimuli.[6] The ubiquity of mobile high-performance computing has now reduced both the size and cost of VR devices, allowing for its use in everyday clinical settings. To date, VR has been used in numerous clinical settings to help treat anxiety disorders, control pain, support physical rehabilitation, and distract patients during wound care.[4, 7–11] For example, VR is effective in decreasing pain during bandage changes for severe burns as an alternative to opioids.[7,12] Similarly, VR reduces pain and provides positive distraction during procedures, such as intravenous line placements[10] and dental interventions.[8]

Our group has previously investigated the role of VR in hospitalized patients. After demonstrating initial feasibility of using VR in the inpatient setting,[13] we conducted a non-randomized, comparative cohort trial comparing the efficacy of a one-time, three-dimensional VR experience versus a two-dimensional nature video in patients with pain.[14] Sixty-five percent of VR patients achieved a clinically significant pain response versus 40% of controls. We found that the effect of VR was independent of the reason for hospitalization or primary cause of acute pain, suggesting that VR may have benefits across conditions. Although our trial was positive, the study was limited by a single, short VR intervention and lack of randomization. Furthermore, we previously documented that existing VR randomized trials have been limited by small sample sizes, uneven methodological quality, and a focus on testing efficacy through formal protocols rather than measuring comparative effectiveness versus active control conditions.[4]

In this study, we performed a comparative effectiveness study evaluating a scalable VR intervention vs. “health and wellness” television programming in a diverse group of hospitalized patients with pain.

## Methods

We conducted a prospective, randomized comparative effectiveness study between November 2016 and July 2017 to compare pain scores of hospitalized patients exposed to either an on-demand, immersive video experience consisting of VR and 360-videos, or an active control consisting of an in-room television tuned to the “Health and Wellness Channel”. We recruited adults aged 18 years or over admitted to the hospitalist, orthopedic, gastrointestinal, or psychiatric consultative services at Cedars-Sinai Medical Center, a large, urban, tertiary care hospital.

**Fig 2:**
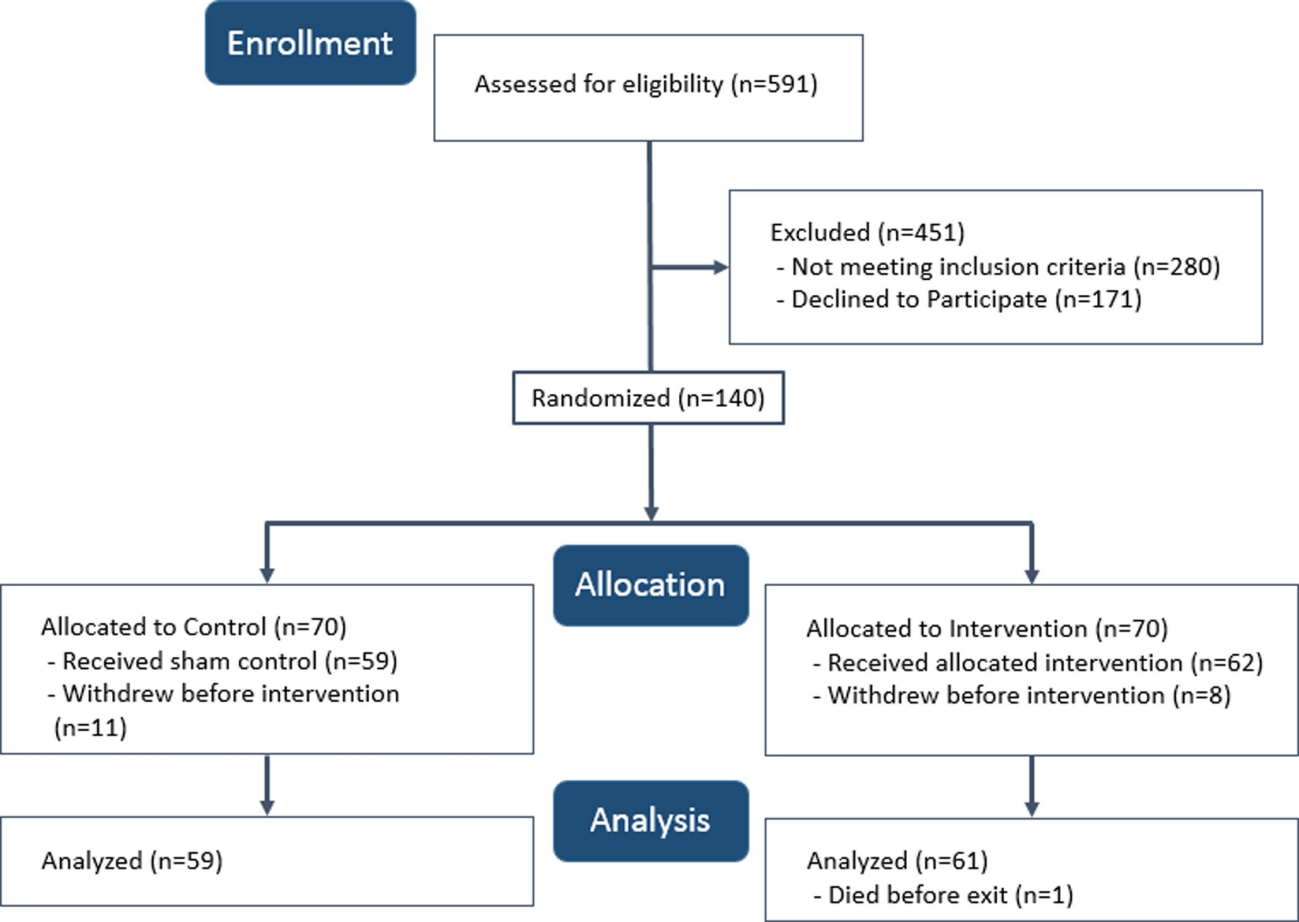
CONSORT diagram describing patient flow through the study.

Patients with an average pain score of ≥3 out of 10 points during the 24 hours preceding patient screening were eligible for inclusion. We chose this pain score cutoff because past studies have found it corresponds to the boundary between mild and moderate pain-related interference with mood and activity.[15] We excluded patients who could not consent or who had head wounds or bandages that may have interfered with the VR headset. In addition, because VR may cause motion sickness in some users,[16] we excluded patients with a history of motion sickness and vertigo and anyone experiencing active nausea or vomiting. **Fig 2 provides** the CONSORT diagram for patient flow through the study.

### Study procedures

Upon providing written informed consent, eligible patients were randomized one-to-one between groups using the Microsoft Excel random number generator. Patients in both groups were informed that researchers were testing the effect of “two types of audiovisual experiences” on the perception of pain. Because it was important for research staff to exhibit equipoise when describing the competing interventions, we prepared a script that used neutral language regarding both interventions. In both arms, we minimized investigator interactions with the study participants, relying on non-study nursing staff to collect pain scores and allowing patients to use their assigned audiovisual experience on their own terms without a formal protocol or order set. In this manner, we designed the study to be a pragmatic assessment of VR compared to an inherent, active control condition already found in the treatment environment, described below.

### Interventions

#### Virtual reality pain distraction experience

**Fig 3:**
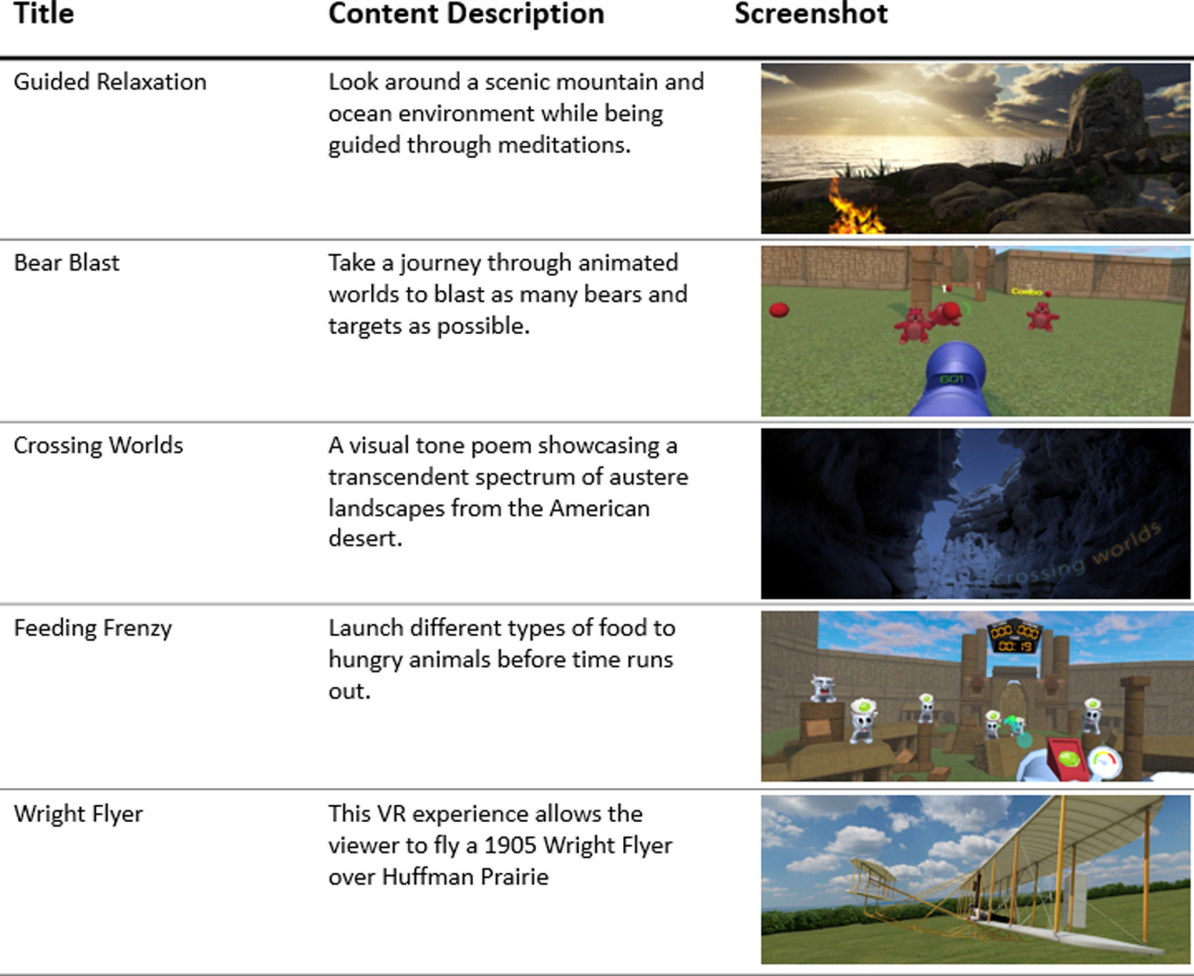
Titles, descriptions, and screenshots of VR experiences available to patients in the experimental group. Complete list of content provided in supplemental materials (S1 Fig). Republished under a CC BY license, with permission from AppliedVR, original copyright 2016.

We administered VR using the Samsung (Ridgefield Park, NJ) Gear Oculus headset fitted with a Samsung Galaxy S7 phone (**Fig 1**). We selected the Samsung Gear because it is commercially available, widely used, relatively inexpensive, has minimal visual latency, and offers an acceptable patient experience based on our previous research.[13, 14] Following randomization to the experimental arm, patients were instructed on procedures for wearing the headset, how to select among twenty-one VR experiences from an application on the phone’s menu (appliedVR; Los Angeles, California), and how to adjust volume and brightness. Patients were asked to use the headset for 10 minutes in the presence of study staff to practice with the equipment, and then advised to use the headsets thrice daily, for 10 minutes per session, and as needed for breakthrough pain over the subsequent 48-hours. Ten minutes was selected to reduce the risk of developing cybersickness, which is a transient sense of vertigo that occurs in a small subset of patients using VR; longer exposure times are associated with higher risk of cybersickness.[17] Following these initial instructions, patients decided for themselves and in partnership with their care team whether, how frequently, and how long to use the VR equipment without direct input from study staff. A complete list of VR and 360-video experiences offered to patients is listed in the supplement (**S1 Fig**), and a partial list is included in **Fig 3**.

We used disposable sanitary covers and foam backing on each headset between patient uses and sanitized the equipment using the protocol described in previous research.[13, 14] **Fig 1** shows an example of the Samsung Gear headset.

#### Control pain distraction experience

To reduce the risk of a Hawthorne effect confined to the VR arm, we employed an active non-pharmacological control condition already present in the hospital environment. Patients in the control arm were instructed to tune their television set to the “Health and Wellness Channel”, which is available in all rooms throughout the hospital. The programming includes guided relaxation content (e.g. yoga and meditation programming), discussions about health and wellness topics, and poetry readings. We selected the programming as a control condition because there is evidence that offering in-room relaxation programming can reduce pain and psychosocial distress in hospitalized patients.[18–23] Following randomization to the control arm, patients received equivalent instructions for use as provided to the VR group; they were asked to view the programming for 10 minutes in the presence of study staff, and then advised to view the channel thrice daily, for 10 minutes per session, and as needed for breakthrough pain.

### Primary outcome

The primary outcome was pain intensity collected via repeated measures in the course of usual care by hospital staff. At three-to-four hour intervals during waking hours, subjects were asked by their assigned nurse to rate their pain using a standard 11-point numeric rating scale (NRS), where 0 is “no pain” and 10 is “worst imaginable pain.” The 11-level pain NRS is supported by the Initiative on Methods, Measurement, and Pain Assessment in Clinical Trials (IMMPACT)[24] as a valid and reliable measure of patient-reported pain. Meta-analysis reveals broad use of the NRS across patient populations with strong evidence of construct validity.[25]

### Secondary outcomes

#### Satisfaction with audiovisual experiences

At the conclusion of their participation in the study, subjects were asked “Would you recommend the audiovisual experience you received here in the hospital to your family and friends?” Responses were collected on a 5-point Likert-type scale ranging from “Definitely Not” to “Definitely Yes”.

#### Hospital consumer assessment of Healthcare Providers and Systems (HCAHPS)

We measured relevant aspects of global patient satisfaction using four selected items from HCAHPS collected from participants 5-weeks post discharge. Two of these items are conceptually related to pain: item 13 of HCAHPS, which reads “During this hospital stay, how often was your pain well controlled?’; and item 14, which reads, “During this hospital stay, how often did the hospital staff do everything they could to help you with your pain?”. Two other HCAHPS questions measured general satisfaction: item 21, which reads, “Using any number from 0 to 10, where 0 is the worst hospital possible and 10 is the best hospital possible, what number would you use to rate this hospital during your stay?”; and item 22, which reads, “Would you recommend this hospital to your friends and family?”

### Opioid usage

Opioid usage was defined as mean total milligrams of morphine equivalent (MME), calculated by first multiplying the quantity of each prescribed medication by the strength of that medication (milligrams of given opioid per unit dispensed), and then multiplying this quantity-strength product by conversion factors derived from published sources to estimate the milligrams of morphine equivalent to the opioids dispensed in the prescription. The mean pre-intervention MME for subjects in each arm was calculated by adding the morphine equivalents for each prescription dispensed during the 48 hours before intervention, while the post-intervention MME for subjects in each arm was calculated by adding the morphine equivalents for each prescription dispensed during the 48 hours after intervention.

### Statistical analysis and sample size

We calculated descriptive statistics for demographic and clinical characteristics of the sample including age, sex, race, ethnicity, primary reason for hospitalization, and baseline pain scores. We performed bivariate analyses to evaluate for significant differences between groups, using two-sample t-tests for continuous variables and chi-square tests for categorical variables.

For the primary outcome, we first compared within-subject differences in immediate pre- and post-intervention pain scores between groups to evaluate the initial impact of the first treatment session, similar to our previous non-randomized trial,[14] using two-sample t-tests and linear regression analysis. Then, we extended the time period by comparing pain-scores recorded during the 48- and 72-hour periods pre- and post-intervention by study group. Because this aspect of the study featured a repeated measures design, and recognizing the within-subject nature of time-series data, we conducted multilevel linear mixed models with pain scores grouped at the subject level as the dependent variable. These regressions included a factor for time, a factor for study group, and a term capturing the interaction between the study group and post-intervention period, isolating the effect of intervention. With only one random effect (subject identifier), we employed an *identity* covariance structure. The control variables (e.g. age, sex, and pain-type) were time-invariant and therefore included in the fixed-effects portion of the model only. To test the appropriateness of using mixed models for these data, we performed likelihood ratio tests comparing ordinary least square and mixed models.

We compared satisfaction with audiovisual experiences between groups using t-tests. We compared HCAHPS item scores between groups using chi-square tests for four-level responses and t-tests for responses collected on the 11-point scale. We compared mean pre- and post-intervention MME between groups using t-tests.

Using power calculations based on a predicted mean VAS pain score of 5.4 (SD 2.7), assuming a change in pain score of 1.5, and targeting a power of 80% (alpha = .05 for two-sided tests), we calculated a total study sample required of 104 using the Stata .power twomeans command.[14] We expanded the sample to 120 in order to accommodate multilevel regressions on repeated measures outcomes that would likely display autocorrelation. All analyses were conducted using Stata 14 (StataCorp).

### Approval

The Cedars-Sinai Institutional Review Board approved this study (IRB Pro00045641) and it was registered with ClincialTrials.gov (NCT02887989).

## Results

### Patient characteristics

**Fig 4:**
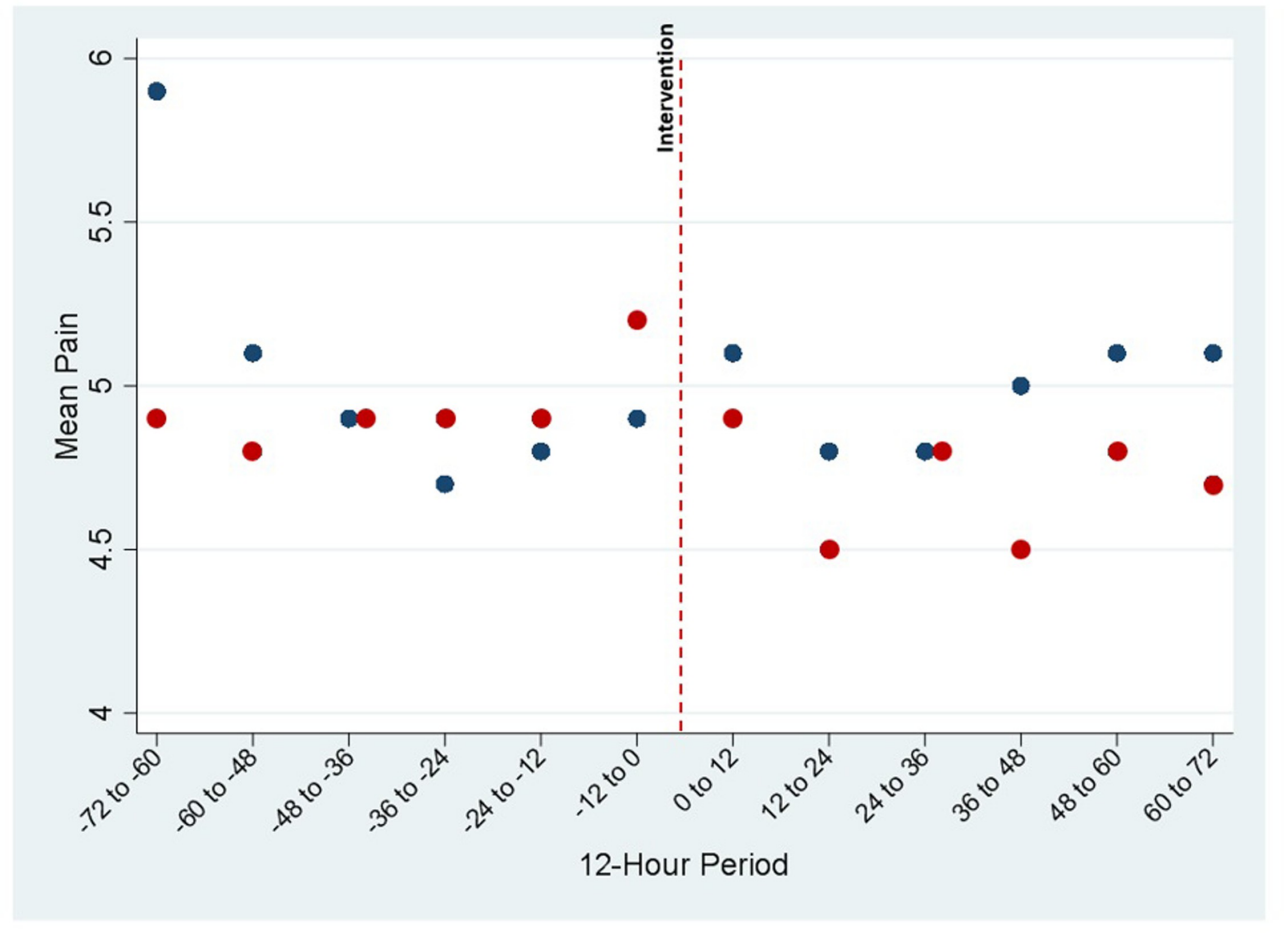
Mean pain-scores for each group at 12-hour intervals during 72-hours before and after the intervention.

One hundred twenty eligible patients completed the protocol, with 61 patients in the VR arm and 59 patients in the control arm. **Table 1** provides baseline demographic and clinical characteristics for the two groups. There were no significant differences between groups for age, sex, race, ethnicity, or pre-intervention pain scores. The reasons for admission were similar between groups. The mean cumulative pre-intervention pain scores were not significantly different between the groups, nor was the mean of the last pain measurement taken pre-intervention. Mean pain-scores for each group at 12-hour intervals during the 72-hours before and after the intervention are displayed in **Fig 4**. We report usage data for the intervention period in the supplement (**S2 Fig**).

**Table 1:** Participant characteristics by study group.

	Controls (n = 59)	VR (n = 61)
Age (SD)	50.0 (15.9)	51.6 (15.1)
Sex, No. (%)		
Male	30 (50.8)	30 (49.1)
Female	29 (49.2)	31 (50.9)
Race, No. (%)		
white	39 (66.1)	38 (62.3)
African-American	10 (17.0)	21 (34.4)
other	10 (17.0)	2 (3.3)
Ethnicity, No. (%)		
Hispanic	11 (18.6)	8 (13.1)
non-Hispanic	48 (81.7)	53 (86.9)
Pain Type, No. (%)		
Visceral	20 (33.9)	21 (34.4)
Somatic	39 (66.1)	40 (65.6)
Service Type, No. (%)		
GI	4 (6.8)	9 (14.8)
Infectious Disease	6 (10.2)	9 (14.8)
Internal Medicine	11 (18.6)	14 (22.9)
Oncology	7 (11.9)	3 (4.9)
Orthopedics	20 (33.9)	16 (26.2)
Other	11 (18.6)	10 (16.4)
Baseline Pain-Score		
≤4	13 (22.0)	16 (26.2)
5–6	20 (33.9)	17 (27.9)
7–8	20 (33.9)	21 (34.4)
≥9	6 (10.2)	7 (11.5)

### Primary analyses

#### Difference in pain scores

The distribution of the within-subject differences in immediate pre- and post-intervention pain scores was tested for normality using the Shapiro-Wilk test (P = 0.19). A T-test showed the mean difference significant in favor of VR (P < .04). When limited to the subgroup of patients with the most severe baseline pain (≥7 points; N = 54), the effect of VR was more pronounced vs. control (-3.04, SD 3.75 vs. -0.93 points, SD 2.16; P = .02). After adjusting for age, gender, and type of pain using linear regression analysis (**Table 2**), exposure to VR conferred a 1.17-point incremental reduction in pain vs. controls (P = 0.048). Age was also a significant predictor in this multivariable model, with each additional 10-years of age predicting a 0.6-point reduction in pain (P = .001).

**Table 2:** Multivariate linear regression on difference between baseline and first post-intervention pain scores (i.e. first post-intervention pain score − baseline pain score) (n = 120).

	β (95% CI)	SE	*P*-value
VR	-1.17 (-2.32, -.01)	.58	.048
Age	-.06 (-.10, -.03)	.02	.001
Sex			
Male	.26 (-.90, 1.43)	.59	.66
Female	Ref		
Pain Type			
Somatic	-.31 (-1.54, .92)	.62	.62
Visceral	Ref		
Prob>*F*	.004		
Adjusted R^2^	.09		

When extending the analysis to include pain scores collected in the 48- and 72-hour periods before and after the intervention, the multilevel mixed model regression analyses (**Table 3**) found VR was associated with significant drops in pain for each period when adjusting for time, study group, age, gender, and type of pain. Models describing the 48- and 72-hour post-intervention periods found .59 (P = .03) and .56 (P = .04) incremental reductions in pain versus controls, respectively.

**Table 3:** Multilevel linear mixed models with pain scores grouped at the subject level as the dependent variable. Independent variables included a factor for time, a factor for study group, and a term capturing the interaction between the study group and post-intervention period that isolated the effect of intervention.

	± 48Hours (n = 120)				± 72Hours (n = 120)			
	β	P>|z|	95% CI		β	P>|z|	95% CI	
Age	-0.04	<0.01	-0.05	-0.02	-0.04	<0.01	-0.05	-0.02
Sex								
Male	-0.00	0.99	-0.61	0.61	-0.01	0.96	-0.63	0.60
Female	Ref	Ref	Ref	Ref	Ref	Ref	Ref	Ref
Pain Type								
Somatic	-0.36	0.27	-1.01	0.28	-0.22	0.52	-0.87	0.44
Visceral	Ref	Ref	Ref	Ref	Ref	Ref	Ref	Ref
Time	0.00	0.50	0.00	0.00	0.00	0.57	0.00	0.00
Group								
VR	0.25	0.44	-0.39	0.90	0.21	0.54	-0.45	0.86
Control	Ref	Ref	Ref	Ref	Ref	Ref	Ref	Ref
Pre/Post								
Post	0.02	0.94	-0.42	0.45	0.06	0.81	-0.41	0.53
Pre	Ref	Ref	Ref	Ref	Ref	Ref	Ref	Ref
**VR-Post Interaction**	-0.59	0.03	-1.13	-0.06	-0.56	0.04	-1.09	-0.03
Observations	5,094				6,680			

All likelihood ratio tests confirmed the appropriateness of employing a mixed model approach.

### Secondary analyses

#### Difference in satisfaction with audiovisual experiences

Among survey respondents, patients in the VR group were significantly more satisfied with their audiovisual experience than patients in the control group (3.5, SD .65 vs. 2.5, SD 1.17; P<0.001).

#### Difference in HCAHPS item scores

Global measures of patient satisfaction, as recorded with selected HCAHPS items administered after discharge, exhibited a ceiling effect in both groups. Patients across the trial were broadly satisfied with their hospital stay upon 5-week reflection, rendering it difficult to identify incremental differences between the experimental groups. Specifically, there were no differences in perceived pain control (P = .48), efforts of the staff to manage pain (P = .42), overall perception of the hospital (P = .69), and willingness to recommend the hospital to a friend (P = .31); scores across all these HCAHPS items were high in both groups.

#### Difference in opioid prescribing

There was no difference in the quantity of opioids consumed between groups in either pre-intervention or post-intervention periods. The mean MME in the VR vs. control groups pre-intervention was 80.83 (SD 51.82) and 75.07 (SD 52.78), respectively (P = .57), and the mean MME in the VR vs. control groups post-intervention was 81.04 (SD 45.09) and 77.08 (SD 43.94), respectively (P = .66).

### VR adverse event monitoring

There were no significant treatment-related adverse events reported in either group. Three patients in the VR group (4.9%) reported transient dizziness at some point during their VR therapy, and all these individuals reported symptomatic resolution upon removing the headset without lasting effects.

## Discussion

Although previous research has demonstrated therapeutic benefits of VR for pain, there has been no prospective, randomized, adequately powered, pragmatic trial of VR versus an active control in hospitalized patients. In this study, we found that on-demand use of VR in a diverse group of hospitalized patients was well tolerated and resulted in statistically significant improvements in pain versus a control group exposed to an in-room “health and wellness” television channel. These results build upon earlier studies and further indicate that VR is an effective adjunctive therapy to complement traditional pain management protocols in hospitalized patients.

Notably, the VR group achieved improved pain scores despite the pragmatic and comparative effectiveness design of the study. Specifically, the trial minimized investigator interactions, did not enforce a VR usage protocol beyond initial patient instructions, relied solely on non-study nursing staff to collect the primary outcome measure, and utilized a control intervention with potential for therapeutic benefits. Moreover, the trial enrolled hospitalized patients with all forms of somatic and visceral pain, including oncologic, neurological, orthopedic, and gastrointestinal pain, among other types. Many of the patients suffered from complex, multi-factorial causes of biopsychosocial distress and received multi-modal treatments, making it difficult for any single intervention to offer consistent pain benefits across this diverse, hospitalized patient population. Nonetheless, the VR intervention achieved statistically significant benefits both initially and after 48-hours and 72-hours of use. Patients also reported higher satisfaction with the VR experience than watching television, indicating an improvement over the current standard of care for in-room audiovisual engagement.

Although the effect of VR was statistically significant, the absolute reduction in pain scores was relatively small. After multivariable adjustment, VR accounted for an incremental 1.17-point drop in pain compared to the control group after the initial treatment; the incremental benefit dropped to 0.59 points when evaluating cumulative pain scores over the subsequent 48-hours. These differences fall below the 2-point threshold for a minimal clinical important difference (MCID) on the NRS, as reported by Farrar and colleagues.[25] However, other studies have established that the MCID is closer to a 13% change from baseline (approximately 1.4 points on a 0–10 scale) [26, 27], which approximates the change observed in this study. Nonetheless, the relatively small effect may have resulted from the pragmatic design of the study, lack of enforced usage protocols, inclusion of clinically diverse patients, and use of an active control that also had potential to contaminate the intervention group. Of note, VR was especially effective in the subgroup of patients with the most severe baseline pain scores (≥7 points), with an incremental benefit of 3.04 points–a value that considerably surpasses the MCID and suggests that VR might be optimal in severe pain. Future research should further explore the differential benefits of VR across patients with varying degrees of pain.

We did not observe a difference in opioid prescribing between the study groups. This is not altogether surprising, as treating physicians were free to manage pain according to usual practice and the protocol did not specify whether or how VR should impact clinical decision-making. Pain medications are typically ordered upon admission, and nurses work with their patients to dispense analgesics according to on-demand need within the constraints of physician orders. Our pragmatic study did not instruct nurses on whether to substitute VR for opioids or any other analgesic. Nonetheless, it is notable that patients in the VR group had lower pain scores despite receiving an equivalent MME as the control group. Future research should evaluate structured and standardized order sets that explicitly interpose VR as an early option prior to initiating or escalating opioids; this may promote earlier and more frequent use of VR and has potential to reduce subsequent use of opioids and other analgesics.

It is notable that despite evaluating 591 patients for participation, only 120 enrolled and completed the protocol. Although this study is, to our knowledge, the largest randomized trial of inpatient VR for pain management, it is important to emphasize the drop-off between patient identification and study completion. This result is consistent with our previous research using VR in hospitalized patients[13, 14] and emphasizes that many patients are ineligible or uninterested in using novel health technologies, such as VR, while hospitalized. Among those who were eligible for the trial, many did not choose to participate for a wide variety of reasons. Patients expressed varying degrees of skepticism, fear, sense of vulnerability, concern regarding psychological consequences, or simply not wanting to be bothered by using the equipment. We believe it is important for the digital health community to recognize that despite the great promise of health technology, clinical realities can undermine expectations.

It remains unknown exactly how VR works to reduce pain perception and experience or whether different forms of VR have varying efficacy. Most proposed mechanisms attribute the benefit to simple distraction.[6] When the mind is deeply engaged in an immersive experience, it becomes difficult to perceive stimuli outside of the field of attention.[28] By overwhelming the visual, auditory, and proprioception senses, VR is thought to create an immersive distraction that restricts the brain from processing pain.[6] Investigators like Hoffman[5, 12, 29, 30], Rizzo[31], Rothbaum[32–34], and Bordnick[35–37], among others, are studying the neurobiological mechanisms of VR across a range of conditions.[4, 11]

Nonetheless, important unanswered clinical questions include: (1) does enhanced VR that applies principles of Acceptance and Commitment Therapy (ACT) such as mindful meditation and/or physiologic biofeedback outperform conventional VR that employs simple distraction? (2) Are there patient characteristics that predict enhanced response to VR beyond baseline pain severity? (3) Are there usage patterns or engagement characteristics that predict enhanced response to VR? (4) Can VR reduce pain while also reducing opioid requirements? Although the current study further supports the effectiveness of VR for managing inpatient pain, it also raises additional questions that deserve inquiry as the field of therapeutic VR broadens and evolves.

## Supporting information

S1 Fig Titles, descriptions, and screenshots of VR experiences available to patients in the experimental group. Republished under a CC BY license, with permission from AppliedVR, original copyright 2016.
(PDF)

S2 Fig Usage data for the intervention period. (PDF)

S1 Doc RCT CONSORT checklist. (DOC)

S2 Doc Study protocol. (PDF)
