# Supplementary material for: Virtual reality for management of pain in hospitalized patients: A randomized comparative effectiveness trial
Source: PLoS One. 2019 Aug 14;14(8):e0219115. doi: 10.1371/journal.pone.0219115 (PMC6693733; doi:10.1371/journal.pone.0219115)
Supplement: S1 Fig — Republished under a CC BY license, with permission from AppliedVR, original copyright 2016. (PDF) [file pone.0219115.s001.pdf]

Title	Content Description	Screenshot
Guided Relaxation	Look around a scenic mountain and ocean environment while being guided through meditations.	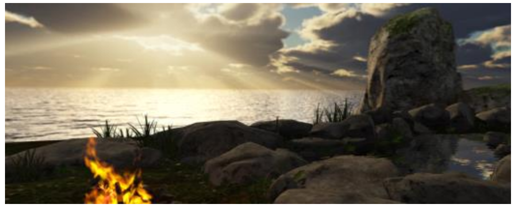
Bear Blast	Take a journey through animated worlds to blast as many bears and targets as possible.	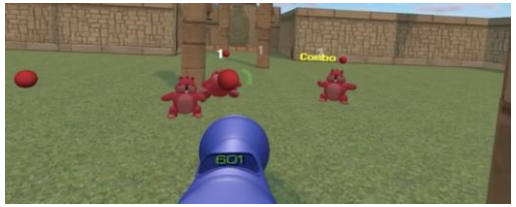
Crossing Worlds	A visual tone poem showcasing a transcendent spectrum of austere landscapes from the American desert.	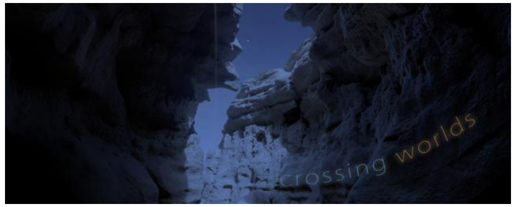
Feeding Frenzy	Launch different types of food to hungry animals before time runs out.	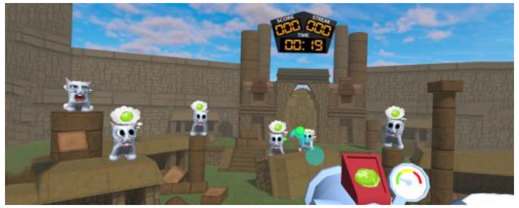
Wright Flyer	This VR experience allows the viewer to fly a 1905 Wright Flyer over Huffman Prairie	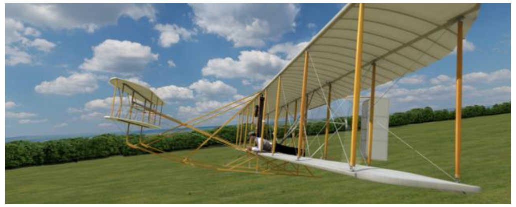

Title	Content Description	Screenshot
Germ Buster	Germs have taken over every room of your beloved home. Fight back with your hydrolaser bubble gun.	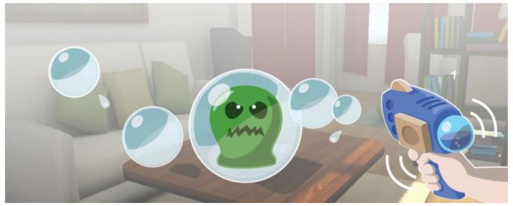
Farm Sanctuary	Transport to the rolling hills and green pastures of Farm Sanctuary's Watkins Glen animal shelter.	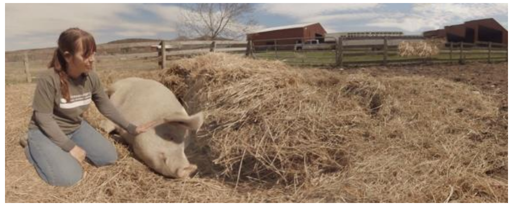
Shape Your Path	Match colored bricks while avoiding obstacles to reach a destination.	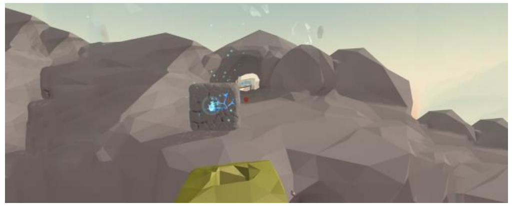
Dream Beach	Relax with beautiful ocean views while listening to the sounds of nature.	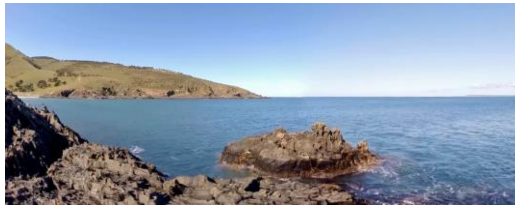
Wild West	Mingle with a group of wild animals in some of the last wild spaces in the West.	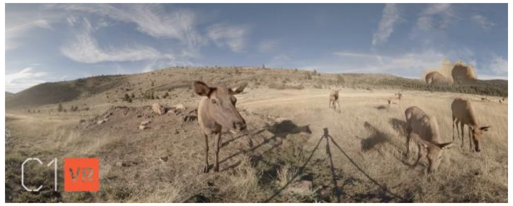

Title	Content Description	Screenshot
The Body VR	Enter the body and learn how the organelles work together to fight deadly viruses.	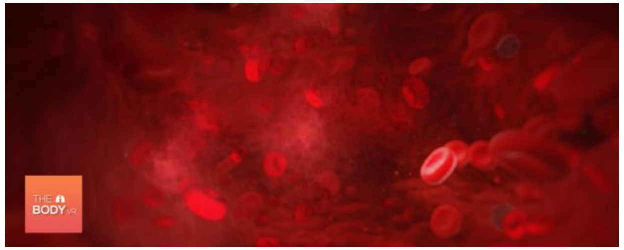
Seals Healing	Dive into a hospital for seals who are released back into the wild when healed, but not before giving you a kiss.	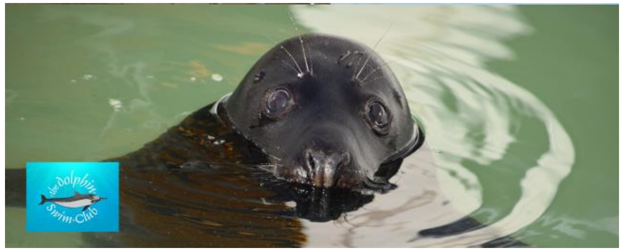
Dolphins Healing	Swim underwater with wild dolphins in their natural, serene home.	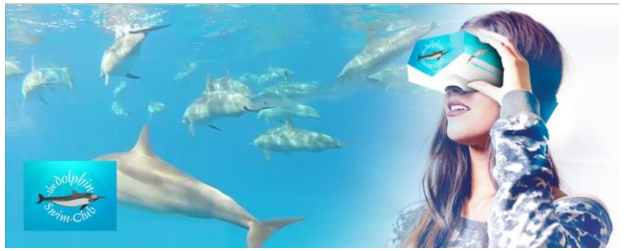
The Art of the Dunk	Put yourself right on the court with the best dunkers in the world.	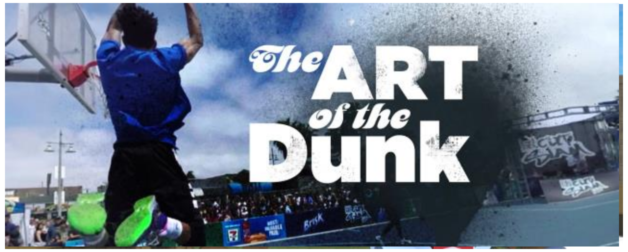
The Redwoods	Take a tour and stand beneath the majestic California Coast Redwoods	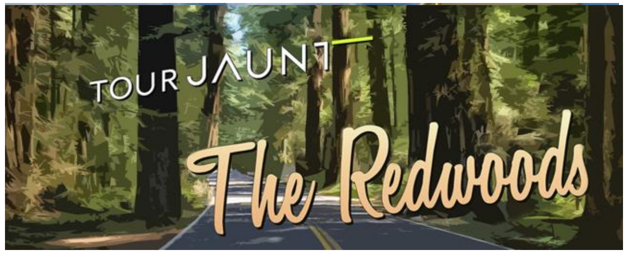

Title	Content Description	Screenshot
Machu Pichu	A guided journey to the mysterious Machu Pichu	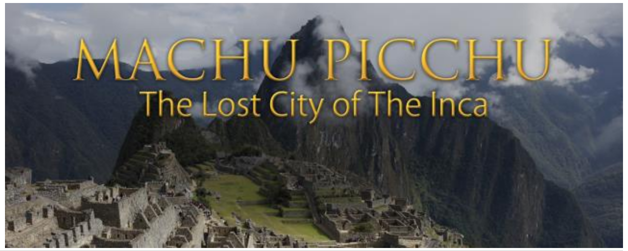
Home Turf: The Needles	Retreat to the Needles with Alex Honnold, one of the world's best rock climbers.	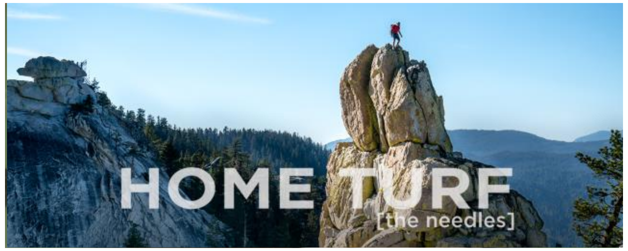
Home Turf: Moab	Walk a tightrope across a canyon in Moab, Utah with slackliner Andy Lewis.	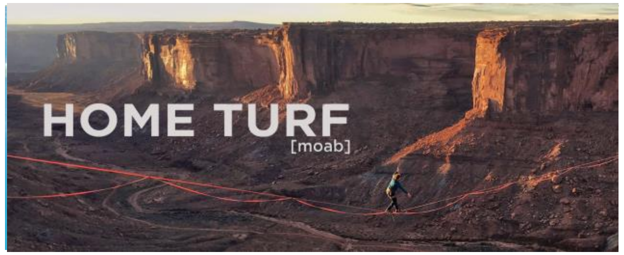
Iceland	Fly over and take in views of Iceland with an exciting helicopter tour.	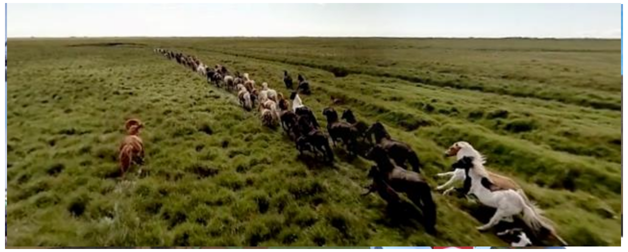
Do Not Push the Red Button	The world's first animated 360-degree Rube Goldberg Machine.	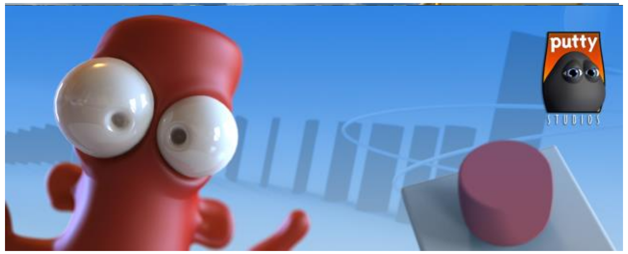
