# Supplementary material for: Virtual reality for management of pain in hospitalized patients: A randomized comparative effectiveness trial
Source: PLoS One. 2019 Aug 14;14(8):e0219115. doi: 10.1371/journal.pone.0219115 (PMC6693733; doi:10.1371/journal.pone.0219115)

S2 Fig presents results by day of an audio-visual usage survey question ("How much of your time today was spent watching TV/Virtual Reality?).

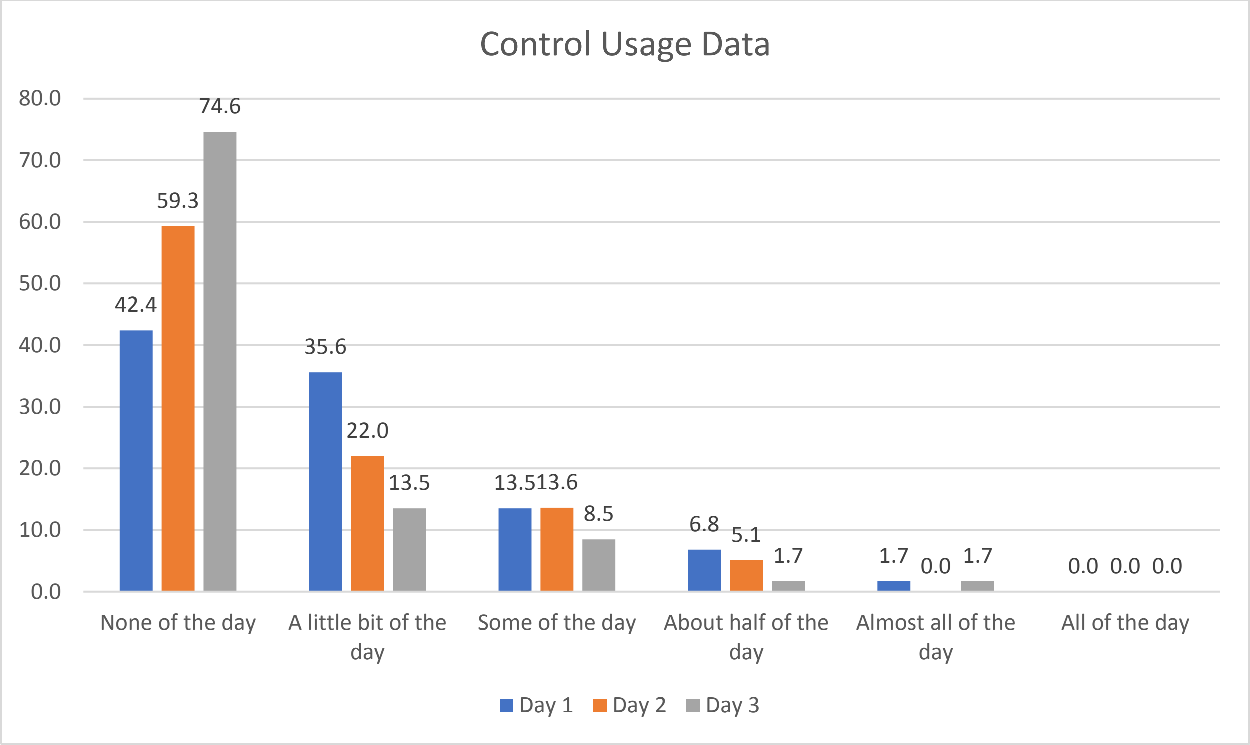

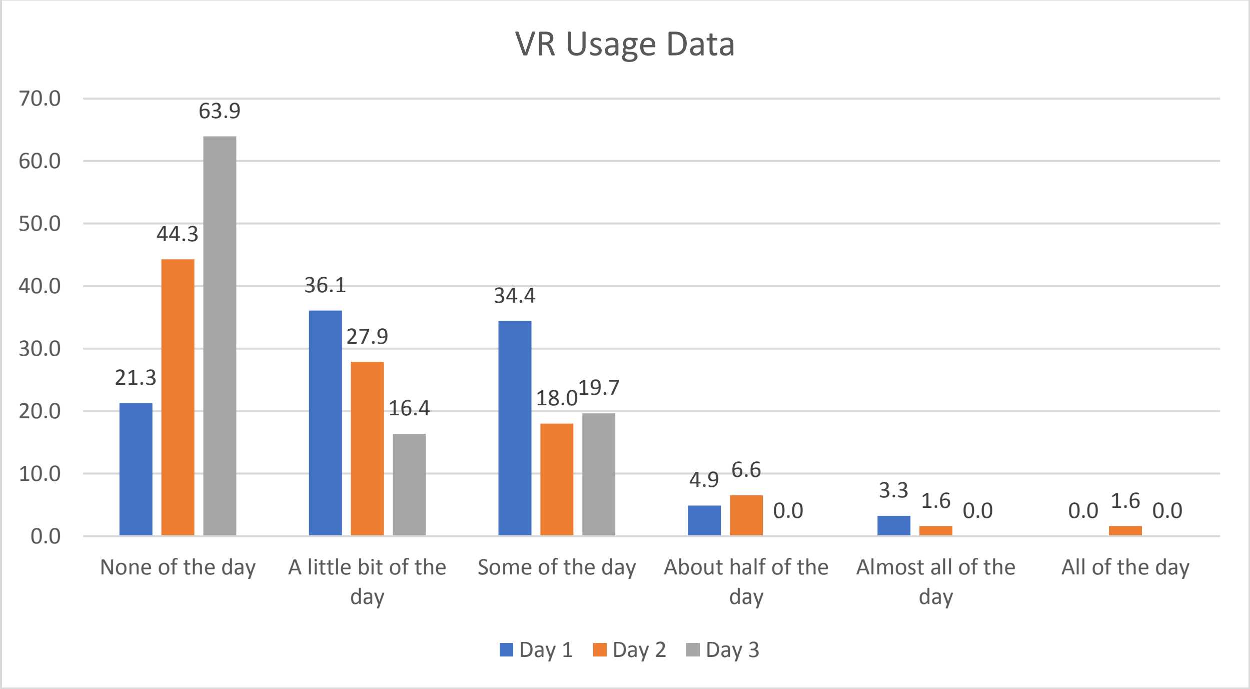

Supplement: S2 Fig — (PDF) [file pone.0219115.s002.pdf]
