# Supplementary material for: Virtual reality for management of pain in hospitalized patients: A randomized comparative effectiveness trial
Source: PLoS One. 2019 Aug 14;14(8):e0219115. doi: 10.1371/journal.pone.0219115 (PMC6693733; doi:10.1371/journal.pone.0219115)
Supplement: S2 Doc — (PDF) [file pone.0219115.s004.pdf]

Study Title: Immersive Virtual Reality Intervention for Non-Opioid Pain Management: A Randomized Controlled Trial

IRB Protocol Number: Pro00045641

Document Date: 11?20/17

ClinicalTrials.gov ID: NCT02887989

Principal Investigators: **Brennan Spiegel, MD**

Bradley Rosen, MD

Other Investigators: **Christopher Almario, MD**

Fransisco Arabia, MD

Itai Danovitch, MD

Fardad Esmailian, MD

Waguih IsHak, MD

Mark Reid, PhD

Alfredo Trento, MD

Odelia Cooper, MD

Sponsoring Institution: **Cedars-Sinai Medical Center**

8700 Beverly Blvd.

Los Angeles, CA 90048

Immersive Virtual Reality Intervention for Non-Opioid Pain Management: A Randomized Controlled Trial

Background:

For decades, inpatient healthcare providers have recognized that psychological and environmental factors influence patients' experience of distress. Pain related beliefs, self-efficacy, sense of control, and coping skills strongly impact perceptions of pain, and psychological interventions targeting these domains improve pain outcomes. VR offers an ability to temporarily alter the way patients experience their environment, facilitating distraction from symptom triggers, increasing sense of control, and providing a safe coping mechanism. By providing an alternative to excess opioid use for breakthrough pain, VR may not only ameliorate acute symptoms, but also diminish opioid related adverse effects such as overdose and opioid use disorder, and reduce hospital length of stay.

VR offers immersive, realistic, three-dimensional experiences that "transport" users to novel environments. Thus, VR has potential to alleviate negative aspects of care by providing multi-sensory information and allowing patients to "escape" to pleasant locations and realities when they are most distressed.

VR has previously been tested in obesity[1-3], neurorehabilitation[4-6], anxiety[7-10] and pain management[11-18]. For example, a meta-analysis of exposure therapies for anxiety disorders – e.g. PTSD, agoraphobia, panic disorder, social anxiety – reported large effects of VR on symptom reports at therapy termination.[10] Similarly, a meta-analysis of post-stroke VR interventions found that patients experience reduced impairment and improved motor functioning with VR compared to usual care.[6] In chronic pain, VR yields benefits for neck pain, procedure-related pain, and for cancer-related pain.[11] In burn care there is a greater reduction in pain and anxiety during bandage changes when patients use VR vs. pain medicines alone.[12] Also, burn patients report lower pain ratings and increased range of motion during physical therapy after immersion in a VR "snow world" that evokes a sense of cold to counteract the pain of a burn.[13]

In addition, the mediators of pain and stress are tied to activation of the hypothalamic-pituitary-adrenal (HPA) axis. Studies of experimental pain as well as acute and chronic pain demonstrate HPA activation through increases of circulating cortisol levels (McEwen BS, Metabolism: Clin and Experimental, 2010; Zimmer C, Clin Journal of Pain, 2003). Further, interventions to reduce pain can lead to decreases in cortisol levels. In one study of patients undergoing laparoscopic cholecystectomy randomized to exposure to music or no music, cortisol levels decreased in the music group (Graversen M, 2013). Similar findings were shown in a study of subjects undergoing colonoscopy (Uedo N, Hepatogastroenterology 2004). Another study demonstrated that chewing gum reduced salivary cortisol levels during a stress evoking procedure (Scholey A, Physiol Behav 2009).

With use of the virtual reality technology to reenact realistic experiences, the role of the HPA axis in pain pathways can be further studied. Administration of the Tier Social Stress Test (TSST) through virtual reality evokes a physiologic stress response in subjects and subsequent elevations in cortisol levels (Fallon MA, Psychosomatic Medicine 2016). Salivary cortisol levels may increase up to 30% on a TSST-VR intervention (Kelly O. 2007. Cyberpsychology & Behavior; Shiban Y. 2016. International Journal of Psychophysiology). However, not all studies confirm changes in cortisol (Montero-Lopez E. 2016. Behav Research; Kotlyar M. 2008. Psychophysiology). Mixed findings may be due to differential response of subjects classified as responders compared to nonresponders and the stressors used (Shiban 2016). Use of virtual reality to reduce endocrine biomarkers of acute pain has not been studied in a controlled setting.

However, despite all the evidence and recent media attention surrounding VR, there have been no controlled trials using VR at scale to manage inpatient pain. Our own work in VR has produced promising results consistent with previous VR research. We recently evaluated the feasibility of delivering VR to inpatients via a "Virtualist" consultation service, finding that VR was acceptable, particularly in younger patients.[19] Further, we recently demonstrated that short-term exposure to a VR interventions called "Pain RelieVR", developed by our partner organization (AppliedVR, Los Angeles, CA, USA), reduced reported pain scores by 24% pre- versus post-intervention across diverse surgical and medical inpatients.[18] A sub-group analysis found that VR was effective for visceral pain from acute digestive disorders, including pancreatitis.[20]

Taken together, this body of research suggests that VR interventions could be deployed as an adjunct to reduce opioid prescription for pain relief, and in some cases as an alternative to opioids. The suitability of the technology for use in a medical setting has been established by our own work, showing that VR is well-tolerated and enjoyed by patients.[18] However, it remains untested if VR can improve patient and resource utilization outcomes at scale across diverse hospitalized patients in a controlled trial. Further, the effect of acute reductions in pain on endocrine biomarkers is unknown.

We will conduct a randomized controlled trial (RCT) of VR non-opioid management vs. a control "sham" intervention for a broad and representative group of medical and surgical patients with pain. Hospitalized patients will receive specialized VR interventions, administered via Samsung Gear headsets, to manage breakthrough pain. Control patients will view content on the in-room Health and Wellness television channel. We will follow patients throughout the course of their hospitalization and monitor outcomes during and after their stays. In addition, we will measure endocrine markers of stress as an optional procedure. Our multidisciplinary team of experts in VR, digital health science, mental health, and pain management will test an intervention that, if effective, could be used in resource-limited environments and serve as an adjunct for patients receiving opioids.

Specific Aims and Hypotheses:

The proposed RCT has the following aims:

1. Assess the relative benefits of using VR versus a control intervention for pain management – including pain scores, usage of pain medication, amount of time between pain medication requests, and complications from opioid pain medication – in a large cohort of hospitalized medical and surgical patients with pain.
2. Assess other characteristics of the hospital stay potentially affected by use of VR usage, including length of stay (LOS), and readmissions.
3. Assess patient satisfaction with general care and pain management following VR usage.

We hypothesize the following regarding these aims:

1. Relative to the control intervention, patients who use VR will report meaningfully reduced pain scores, will require less pain medication (opioid equivalents, and non-opioids), will report fewer opioid complications (e.g., nausea, vomiting, constipation), and will delay requests for breakthrough doses of pain medication.
2. Compared to controls, patients who use VR in the hospital will stay in the hospital for less time, report greater satisfaction with care, and will experience fewer readmissions.
3. Compared to controls, patients who use VR will report greater satisfaction with care, as measured by HCAHPS scores after 6 weeks of patient discharge

Exploratory aim:

1. Assess for reduction in cortisol levels with use of VR

Study Procedures:

Overview and Study Design

The study is an RCT that will enroll up to 244 inpatients with diverse diagnoses to test the efficacy of VR for long term pain management as an adjunct to opioid pain medication. We will randomize one group of patients to receive a VR-based intervention, and the other group of patients will receive a control "sham" intervention. We will randomize patients using the MS Excel random number generator to ensure that there is an equal distribution of participants in the control vs. experiment group. We will measure changes in self-reported pain, opioid pain medication usage (including breakthrough pain), time between doses, and other characteristics of the hospital visit. We will also assess patient satisfaction after 6 weeks using the HCAHPS survey. We will assess pre and post changes in pain between both the VR and control groups.

Originally, study participants were selected by an Investigator (Drs. Almario, Arabia, Danovitch, Esmailian, IsHak, Rosen, Spiegel, Lin, Little, Wiss, Moon, or Trento) and any other CSMC physicians who wished to refer their patients who met inclusion criteria. In the next phase of the study when the focus turns to orthopedic patients, CSMC physicians Little and Garlich and any other CSMC orthopedic physician will identify and refer orthopedic patients who meet inclusion criteria to participate in this trial. During the VR Intervention, we will place VR hardware on patients in their rooms, and allow

patients to use VR software for a 10-minute trial run. If they are interested, patients will be allowed to continue to use the VR software as needed for the remainder of their hospital stays. During the control “sham” intervention, patients will be instructed on the use of the in-room television system, and then shown 10 minutes of the Health and Wellness CARE Channel; these patients will be encouraged to repeat viewing as needed during the remainder of their hospital stays.

VR Intervention

As previously described, VR interventions have been used as distractions or diversions during medical procedures. This study will be one of the first RCTs to test deployment of low-cost VR as an adjunct to opioid pain management in an inpatient hospital setting. The VR hardware configuration and all software is currently being developed by AppliedVR, a leader in the development of medical applications for virtual reality technology, including interventions for weight loss and health, safety training, education, and personal development. Details about the VR hardware use can be found at the end of this document (See **VR Manual** in **Additional Information**), and are summarized below.

VR Hardware:

This RCT will utilize a Samsung Gear VR goggle set, fitted with a Samsung Galaxy phone that delivers VR images and sound (Figure 1a). Users will wear the VR goggles as shown in Figure 1b. Study staff will familiarize inpatients with hardware before use, and allow them to test use on their own before leaving the initial visit.

Figure 1. VR Hardware

1a. VR Goggles and Phone

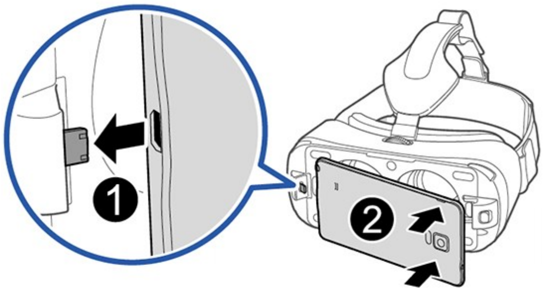

1b. VR Goggles on user

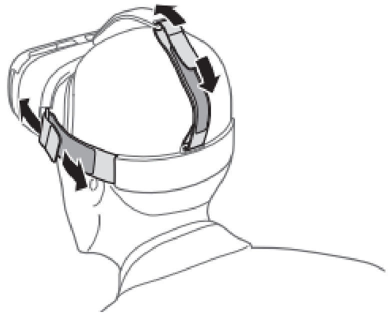

VR Hardware Placement and Cleaning:

The Samsung gear device will be put in place as follows:

1. Patient places sanitary hairnet on head
2. Goggle is fitted with disposable foam backing affixed to inside of face mask
3. Study coordinator places goggle headset on patient and adjusts straps

Between patients, the device will be cleaned as follows:

1. Disposable hairnet on patient and foam backing will be thrown in sanitary waste bin in inpatient room.
2. Fabric strap will be cleaned by coordinator using Virex, approved cleaner for medical fabrics and let sit for a minimum of 10 minutes before next use.
3. Plastic housing of device will be cleaned using Sani-wipes and let sit for a minimum of 2 minutes before next use.
4. Inside and outside of the lenses of device will be cleaned using approved lens cleaner containing alcohol.

VR Software:

In VR, 3D visual and audio cues immerse the patient. Software modules may be used as needed during the inpatient stay for as long as a patient wishes. We are requiring 10 minutes for initial test use in the presence of study staff, and then recommending a maximum of 30 minutes at a time, each followed by 15 minute breaks. Patients will be shown how to select VR experiences from the phone menu (Figure 2).

Figure 2. VR Software Menu

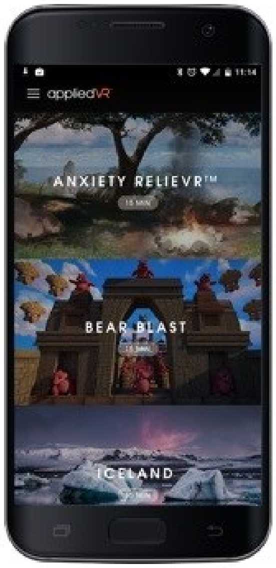

VR interventions include the following (see Figure 3):

Figure 3. VR Environment Examples

- **Iceland Flyover**: Patients fly over photorealistic views of world landmarks, allowing them to visit famous places from the hospital bed.

3a. Iceland Flyover

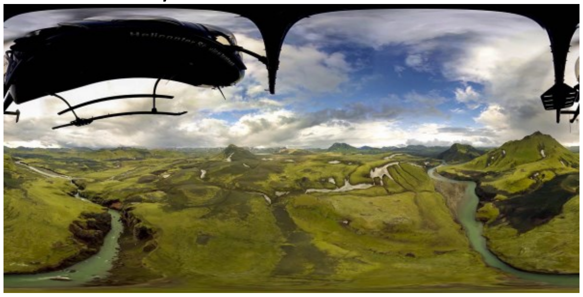

- **Pain RelieVR**: Patients are immersed in a fantasy world where they use their gaze to direct objects at moving targets, while exposed to colorful imagery and soothing music.

3b. Pain RelieVR

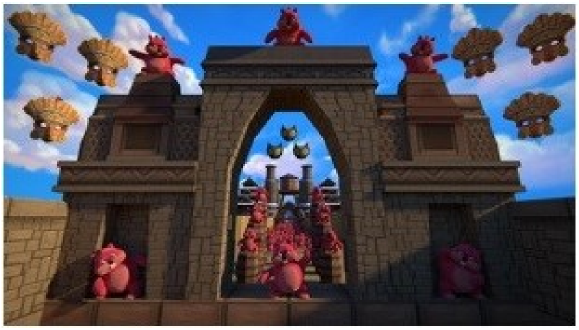

- **Anxiety RelieVR**: Patients experience an interactive, meditative landscape along a peaceful shoreline.

3c. Anxiety RelieVR

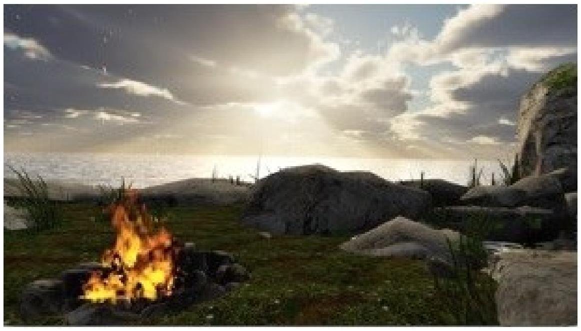

Study Population and Period

The overall study population will consist of up to 244 patients – 122 patients who are exposed to VR, and 122 control patients exposed to an audiovisual "sham" intervention. We will randomize patients using the MS Excel random number generator. We will select up to 244 hospitalized patients at CSMC admitted during the study period of 1 October 2016 to 30 September 2018. The inpatient wards serve patients ranging in age from 18 to over 100, allowing us to assess the feasibility of using VR across diverse age groups. Initially, we did not restrict eligibility to any particular diagnosis, although we will collect this demographic information for subsequent subgroup analysis. All participants were selected at random by the Co-Investigator (Dr. Rosen), by investigators in the gastroenterology service (Drs. Almario and Spiegel), or the Heart Institute (Drs. Arabia, Esmailian, and Trento) or psychiatry service (Drs. Danovitch and IsHak), or any CSMC physician who wishes to refer patients who meet the eligibility

criteria. However, preliminary analysis on patients enrolled in the study from 1 October 2016 to 30 September 2017 indicate that patients with higher baseline pain intensity experience greater reductions in pain as a result of VR when compared to the control group. Upon several subgroup analyses and communications with Cedars-Sinai providers, we determined a change in focus of diagnosis would improve the power of these analyses. The Cedars-Sinai Orthopedic Department has a large and steady supply of high severity pain patients that might benefit from a pain distraction intervention. The department's physicians have also expressed a strong interest in alternative pain treatment modalities and would like to explore these distraction methods further. We will use the existing protocol to continue to recruit only hospitalized orthopedic patients for a full cohort of 160 orthopedic patients for this trial. Because our currently enrolled sample (1 October 2016 to 30 September 2017) already includes some orthopedic patients, we will recruit only additional orthopedic patients until we complete the cohort of 160 orthopedic patients, accounting for possible withdrawal from the study. Once this cohort of patients is complete, the overall sample size for the entire study including patients from all diagnosis groups will be up to 244 patients.

Eligibility Criteria

The following criteria will be used to define patient eligibility:

Inclusion Criteria:

- Able to understand the goals of the study and provide informed consent
- Until 30 September 2017, any hospitalized patient under care of CSMC inpatient service (ISP), gastroenterology, or psychiatry, admitted between 1 October 2016 and 30 September 2017, who is not excluded due to criteria listed below.
- From 1 October 2017 Until 30 September 2018, any hospitalized patient under the care of CSMC orthopedic providers
- At least 18 years of age
- English speaking

Exclusion Criteria:

- Unable to consent to study due to cognitive difficulty
- Current diagnosis of epilepsy, dementia, or other neurological disease that may prevent use of VR hardware and software
- Sensitivity to flashing light or motion
- Pregnancy, or a medical condition where the patient is prone to frequent nausea or dizziness
- Recent stroke
- Post-transplant patient, or pre-transplant patient with severe illness
- Patient on ventilator, BiPAP, or other breathing assistance equipment

- Injury to the eyes, face, neck, or arms that prevents comfortable use of VR hardware or software, or safe use of the hardware (e.g., open sores, wounds, or skin rash on face)
- Non-English speaking
- Use of glucocorticoids during hospitalization
- Pituitary adenoma or sellar mass
- Adrenal masses
- Subarachnoid hemorrhage

Patient Recruitment and Study Procedures

Originally, appropriate inpatients were selected at random by Dr. Rosen or other authorized co-investigators or study staff, who will obtain relevant clinical variables from the EHR of their own patients. All patients admitted to ISP had Dr. Rosen (or a physician under his employ) listed as the Attending Physician of Record for the current hospitalization. Some inpatients approached for this study have received consultation from gastroenterology by Drs. Almario or Spiegel, at the request of the patient's Attending Physician of Record for the current hospitalization. Other inpatients approached for this study have received consultation from psychiatry by Drs. Danovitch or IsHak, at the request of the Attending Physician of Record. Other inpatients were approached by an Attending Physician of Record affiliated with the Heart Institute (Drs. Arabia, Esmailian, and Trento, or others who operate under them) and the Orthopedics department (Drs. Lin, Little, Wiss, and Moon All other CSMC physicians wishing to refer patients will provide a research interest letter to each eligible patient. In the next phase when we restrict the inclusion criteria to orthopedic patients, CSMC physicians Little and Garlich and any other CSMC orthopedic physician who wishes to refer his or her patients for this study will provide eligible patients with information about this research. Once the patient has expressed interest in participating, the patient will then be approached for consent by one of the study personnel. The location and identity of the inpatient will be relayed to a member of the study team, who will deliver a randomly selected intervention (either VR or Health and Wellness CARE Channel) to the patient the same day (at the patient's discretion). The patient will continue usage as needed for up to 20 days of the hospital stay. Patient selection will continue until we have obtained a total of 244 patients, with 122 in each group. Of these 244 total patients across all diagnosis groups, 160 will be orthopedic inpatients.

Experimental Group

Pre-Intervention Procedures:

For patients randomized to the experimental condition, the study staff member will arrive at bedside, explain the risk and benefits of the study, and will obtain informed consent. Patients will be advised that standing puts them at risk for a fall, and that they should remain seated for each VR intervention. Next, the study staff member will explain the use of the Samsung VR goggle set, and thereafter will allow the patient to use the device freely for up to 30 minutes at a time. An interval of 10 minutes is suggested for first-time use, but patients will be allowed to continue as long as they are not experiencing any

discomfort or side effects (dizziness, motion sickness, etc.). The study staff member will be present during the initial visit, and will work with hospital staff as necessary to ensure the patient is receiving appropriate clinical care throughout the duration of the intervention.

Salivary cortisol will be collected as an optional procedure twice, pre and post demonstration of VR and the Health and Wellness channel.

Collection Instructions:

1. Do not brush teeth before collecting specimen.
2. Do not eat or drink for 15 minutes prior to specimen collection.
3. Do not use any creams, lotions or steroid inhalers immediately prior to the collection.
4. Collect specimen and record collection time.
5. To use the Salivette:
    a. Rinse mouth thoroughly with water and discard.
    b. Remove top cap of container to expose swab.
    c. Place swab directly under the tongue until well saturated, approximately 1 minute.
    d. Do not touch swab with fingers.
    e. Place swab back into its container without touching, and replace the cap.
6. Record collection time, and send appropriately labeled Salivette to laboratory.

If the patient expressed interest in keeping the VR hardware longer, and also reports no adverse effects after using the goggles for a 10-minute test run with study staff present, the VR hardware will be left in the patient room. If VR hardware is lost or damaged, patients will be informed that they will not be held responsible. The patient and/or a member of the patient's family will be shown how to use the device as needed, and will also be shown how to recharge the battery if needed. Most patients will use the VR equipment during an interval when they are not receiving clinical care, and could use an entertaining diversion. Study staff will check daily on the status of the patient by contacting the patient's Primary Care Provider. Patients will be asked to log the time and duration of device usage.

Post-Intervention Procedures:

Study staff will retrieve the VR equipment after the patient is no longer interested in using the goggles, the patient is within 24 hours of discharge, or 20 days have elapsed since beginning participation. Pain ratings and medication usage history throughout the hospital will be retrieved from the EHR by authorized study staff for analysis after patient discharge. All patients will be sampled by the HCAHPS Picker survey to assess patient satisfaction.

Control Group

Concurrently with VR intervention participation, we will collect data from up to 122 inpatients (from all diagnosis groups combined) who will serve as controls for this study, selected using the same eligibility criteria used for this study. We will attempt to match control participants based on age, sex, ethnicity, and admitting diagnosis to reduce random variability in the data, but we will still evaluate this as a

between-subjects comparison. These patients will be actively recruited and consented, and they will only interact with study staff for approximately 45 minutes, including time for consent, review of equipment to be used in the intervention, and recording usage time.

Control Group Intervention and Procedures:

Figure 4. Still image from in-room Health and Wellness. Channel

Control participants will be given similar instructions to those who have participated in the standard VR intervention, except they will be exposed to a "sham" intervention that is part of usual hospital care. They will be offered the chance to participate in the study, and if they consent, they will follow the same *Pre-Intervention Procedures* outlined for VR Intervention participants (a physician will collect heart rate and pain data via chart review Then, the patient will be shown how to use the in-room Health and Wellness relaxation television channel – called the Health and Wellness Channel (http://www.healinghealth.com/) . The patient will be encouraged to view the channel for approximately 30 minute intervals during the remainder of their hospital stay. After discharge, the physician will collect heart rate and pain data via chart review.

Study Measures

Pain Ratings

As part of usual care, clinical staff regularly obtain assessments of pain intensity from patients using a Numerical Rating Scale (NRS), usually reported on a 0-10 scale, where 0 is no pain, and 10 is the worst pain imaginable. Previous healthcare studies[21-24] have established that NRS is an acceptable method for unidimensional assessment of pain intensity. In this study, patients will not be asked about pain as part of the study. Instead, pain scores reported to the care provider and entered into the EHR will be used.

VR/TV Usage Monitoring

There is no standardized method for assessing viewing time for VR or other audiovisual experiences, without some form of supervision (e.g., automatic, electronic logging on the viewing device; monitoring by study staff; etc.). Currently, we lack the ability to track both TV and VR viewing times through some automated process, so we will provide patients with activity logs, which they will use to record the start time and duration of any viewing experiences (on TV or VR). We realize this method will introduce reporting error, but this error should be equally distributed across both study groups.

HCAHPS

CSMC collects patient satisfaction data using the HCAHPS Picker survey, administered by a third party. In accordance with regulations guiding this survey of patient care experiences, we will not directly influence responses in any way. Instead, we will sample 100% of all study participants, expecting a

response rate of approximately 25%. We will use these data to estimate changes in satisfaction associated with exposure to the intervention condition, versus the control condition. The HCAHPS survey has demonstrated face validity in previous studies.[28]

Salivary cortisol assay (OPTIONAL):

The salivary cortisol assay will be processed at the lab at CSMC which is then shipped to Quest Diagnostics. It is stable at room temperature for 5 days. The assay is performed using liquid chromatography/tandom mass spectrometry. While cortisol has a diurnal variability with levels highest in the early morning, we will be assessing percent change of cortisol levels over the 10 minutes of the VR demonstration or Health and Wellness channel viewing in each individual. This will minimize the effect of the diurnal variation.

Data Collection

We will collect the following information about VR intervention and Control participants:

- Name and Medical Record Number (secured and stored separate from all other data below)
- Age
- Sex
- Race/Ethnicity
- Admitting Diagnosis during inpatient stay
- ISP ward
- Previous diagnoses of epilepsy and other neurological or visual impairments.
- All Pain ratings throughout the hospital stay.
- All opioid and non-opioid pain medication usage information (dosage amount and timing).
- HCAHPS Patient Satisfaction Picker Survey responses.

Demographic and descriptive clinical data will be abstracted by Dr. Rosen or authorized study staff at the time of patient selection, and vital signs will be collected before and after the intervention.

Analysis Plan:

The current study plan allows us to enroll an average of three patients per week at a minimum.

We will calculate descriptive statistics for patients in each group. We will then perform bivariate analyses to compare mean cumulative pain scores, morphine milligram equivalents, and average length of stay between groups using t-tests. We will compare the proportion with an opioid complication between groups using chi-squared, and calculate the “number needed to treat” in order to prevent one additional complication with VR vs. no usual care. We will then perform multivariable linear regression analysis to predict pain scores while controlling covariates, including age, demographics, comorbidities, and baseline pain scores. We will report both intention-to-treat analyses including all evaluable patients

by randomized allocation, and per-protocol analyses limited to just those patients in the intervention arm who opt to use VR (while accounting for dose-response potential). We will adopt a P-value of <0.05 as evidence for statistical significance, and calculate 95% confidence intervals around point estimates. All analyses will be performed using Stata for Windows (StataCorp, College Station, TX).

We have powered this study based on our previous research on pain management with VR[18-20], which reported clinically meaningful shifts of approximately 1 SD in self-reported pain scores following brief exposure to VR. Several previous studies[21-23] have established that the minimal clinically important difference on patient-reported pain rating scales (visual analog or numerical ratings) is approximately 13% (approximately 1.4 points on a 0-10 scale, with a standard deviation of 14%). Thus, we are interested in measuring a 1SD change in pain ratings following exposure to the VR intervention, but we will collect a sufficiently large sample to measure smaller changes both within patients exposed to VR, and in comparison to a control group. For all analyses, we will assume a 5% Type I error rate (alpha) and a 20% type II error rate (or 80% power). For **within group analyses**, we would require a minimum of 10 patients to detect a 1 SD change in pain scores. However, we have powered the study to detect a 0.38 SD difference **within each group** (with 58 patients per group), however have modified this to 80 patients per group. We used this method to calculate and justify the required sample size for a complete cohort of orthopedic patients. Therefore, the overall intervention and control groups, along with the orthopedic subgroup will be adequately powered for analysis. The sample-size modification is required due to over-estimation of adherence, and aims to support a "per-protocol" analyses. We plan on conducting this per protocol analysis among treatment-compliant subjects *in addition* to the intent to treat analysis. The additional subjects, then, will bring the total N of the per-protocol analysis into a range capable of detecting the hypothesized effect-size used in the original sample-size calculation. While sample-size re-estimation based on observed difference with a limited number of subjects may be biased and misleading, we feel that the goal of the trial (determining efficacy rather than effectiveness) - coupled with the fact that randomization procedures will remain unchanged - supports the request.

For **between group analyses**, we will compare pain scores obtained from all inpatients exposed to the VR intervention to a **80-patient control group**. We expect the variability in pain scores to be comparable to that observed in VR intervention participants (approximately 1 SD change over a 20-minute period, equivalent to an effect size of 1.0), so we are collecting data from the same number of patients (80) with complete data in Phase 2. We have powered this study to detect an SD difference as low as 0.52 between the VR intervention and control participants (requiring 80 patients in each group).

We will conduct an exploratory **subgroup analysis** to detect changes in salivary cortisol between patients exposed to VR vs patients exposed to the control intervention.

As of September 2017, 9 patients have been consented to participate in this sub-study, but only 1 patient yielded sufficient quantity of samples for a pre- and post-intervention cortisol analysis. The majority of samples were of inadequate quantity for laboratory analysis. Given that all participants eligible to participate in the main study are also eligible for this sub-study, each patient consenting to the main study will be asked to participate in the cortisol sub-study. Accounting for attrition and the

technical complexities of cortisol sampling that often results in low yield quantities, we will recruit 113 patients total (including the 9 that have already been consented as of September 2017).

Risks and Benefits:

Benefits:

To Individual: Patients will likely enjoy using the VR interventions, which are immersive and impressive upon cursory use. We anticipate that patients may report greater satisfaction, pain management, and overall health during the study.

To Society: This will be one of the first RCTs conducted on inpatient use of VR for pain management. Knowledge gained could be used to improve patient satisfaction with care and pain management across all hospitals.

Risks:

Patients using the virtual reality intervention may experience side effects common to users of VR and individuals who view 3D video, including motion sickness, dizziness, eye strain, headaches, or other visual abnormalities. If patients experience these symptoms, they will be asked to stop using the VR software for 15 minutes, and will be allowed to continue *if* they agree and wish to proceed. A small number of patients (up to 0.025%) may experience seizures or severe symptoms (e.g., disorientation, nausea, or drowsiness) upon viewing the virtual reality experience. Seizures from flashing light are more common in children and epileptic patients (who are excluded). To minimize this concern further, we have *not* incorporated flashing lights into the VR experiences. Some patients may find the VR goggles uncomfortable to wear or confining. To date, patients with claustrophobia have *not* reported discomfort using VR goggles, as they are often used in treatment of that condition. Nevertheless, individuals previously diagnosed with claustrophobia should discontinue use if they feel uncomfortable. A trained study staff member will be on hand during initial use to ensure patient safety. Patients who use the VR headset over several days are instructed to use it for a maximum of 30 minutes at a time. In pilot testing, patients have not reported any adverse effects from extended use of VR hardware.

Subjects may feel uncomfortable or embarrassed when answering survey questions. If subjects are uncomfortable or embarrassed answering any survey question, they may skip it. The survey will be labeled with a unique study number so that only the research team can recognize subjects.

Data Security and Privacy:

Data will be abstracted from the EHR by approved staff (Dr. Rosen and other authorized personnel), and will be transmitted securely to study staff. Staff data collection will be limited to variables mentioned previously. Demographic and clinical data, as well as Qualitative Interview responses will be entered into a secure database and stored on secure servers at CSMC. We will not collect Social Security numbers,

and we will use sequential Unique ID numbers to track patients in the study. All collected specimens will be de- identified before they are sent out to Quest Diagnostics for analysis.

Conclusion:

We are testing the feasibility of deploying a VR intervention to inpatients at CSMC. We believe this intervention can be used to improve pain management, patient satisfaction, and overall patient health as well as providing insights into endocrine mediators of pain. If successful, we will expand this study to test the effectiveness of this intervention in subgroups of patients who respond with greatest improvements in care ratings as well as lead to more comprehensive studies of the HPA axis in modulation of pain. The study results, including suggestions made by patients during this intervention will also be used to guide the development of tailored versions of the VR environments for future immersion exercises.

References:

1. Manzoni GM, Cesa GL, Bacchetta M, et al. Virtual Reality-Enhanced Cognitive-Behavioral Therapy for Morbid Obesity: A Randomized Controlled Study with 1 Year Follow-Up. *Cyberpsychology, behavior and social networking*. Oct 2 2015.
2. Cesa GL, Manzoni GM, Bacchetta M, et al. Virtual reality for enhancing the cognitive behavioral treatment of obesity with binge eating: randomized controlled study with one-year follow-up. *JMIR*. 2013;15(6): e113.
3. Riva G. The key to unlocking the virtual body: virtual reality in the treatment of obesity and eating disorders. *J Diabetes Sci Tech*. Mar 2011;5(2):283-292.
4. Ogourtsova T, Souza Silva W, Archambault PS, Lamontagne A. Virtual reality treatment and assessments for post-stroke unilateral spatial neglect: A systematic literature review. *Neuropsych Rehab*. Dec 1 2015:1-46.
5. Pedroli E, Serino S, Cipresso P, Pallavicini F, Riva G. Assessment and rehabilitation of neglect using virtual reality: a systematic review. *Frontiers in Behav Neurosci*. 2015;9:226.
6. Saposnik G, Levin M, Stroke Outcome Research Canada Working Group. Virtual reality in stroke rehabilitation: A meta-analysis and implications for clinicians. *Stroke*. 2011; 42: 1380-1386.
7. McCann RA, Armstrong CM, Skopp NA, et al. Virtual reality exposure therapy for the treatment of anxiety disorders: an evaluation of research quality. *Journal of anxiety disorders*. Aug 2014;28(6):625-631.
8. Malbos E, Boyer L, Lancon C. [Virtual reality in the treatment of mental disorders]. *Presse medicale*. Nov 2013;42(11):1442-1452.
9. Rothbaum BO, Garcia-Palacios A, Rothbaum AO. Treating anxiety disorders with virtual reality exposure therapy. *Revista de psiquiatria y salud mental*. Apr-Jun 2012;5(2):67-70.
10. Parson TD, Rizzo AA. Affective outcomes of virtual reality exposure therapy for anxiety and specific phobias: A meta-analysis. *JBehav Therapy and Exper Psych*. 2008; 39: 250-261.
11. Li A, Montano Z, Chen VJ, Gold JI. Virtual reality and pain management: Current trends and future directions. *Pain Management.* 2011; 1:147-157.
12. Hoffman HG, Chambers GT, Meyer WJ, Arceneaux LL, Russell WJ, Seibel EJ, Richards TL, ... Patterson DR. Virtual reality as an adjunctive non-pharmacologic analgesic for acute burn pain during medical procedures. *Annals of Behav Med*. 2011;41(2):183-191.
13. Carrougher G J, Hoffman HG, Nakamura D, Lezotte D, Soltani M, Leahy L, … Patterson DR. The effect of virtual reality on pain and range of motion in adults with burn injuries. *J Burn Care & Res* 2011;30(5), 785-791.
14. JahaniShoorab N, Ebrahimzadeh Zagami S, Nahvi A, et al. The Effect of Virtual Reality on Pain in Primiparity Women during Episiotomy Repair: A Randomize Clinical Trial. *Iranian J Med Sci*. May 2015;40(3):219-224.
15. Hua Y, Qiu R, Yao WY, Zhang Q, Chen XL. The Effect of Virtual Reality Distraction on Pain Relief During Dressing Changes in Children with Chronic Wounds on Lower Limbs. *Pain Manage Nurs.* Oct 2015;16(5):685-691.

16. Malloy KM, Milling LS. The effectiveness of virtual reality distraction for pain reduction: a systematic review. *Clin Psych Rev*. Dec 2010;30(8):1011-1018.
17. Morris LD, Louw QA, Grimmer-Somers K. The effectiveness of virtual reality on reducing pain and anxiety in burn injury patients: a systematic review. *Clin J Pain*. Nov-Dec 2009;25(9):815-826.
18. Tashjian VC, Mosadeghi S, Reid MW, Martinez BM, Ahmed F, Dailey F, Robbins K, Rosen B, Spiegel BMR. The Clinical Utility of Virtual Reality in Inpatient Pain Management. 2016 *In Preparation*.
19. Mosadeghi S, Reid MW, Martinez B, Rosen BT, Spiegel BMR. Feasibility of an Immersive Virtual Reality Intervention for Hospitalized Patients: An Observational Cohort Study. *JMIR Ment Health* 2016;3(2):e28 DOI: 10.2196/mental.5801 PMID: 27349654.
20. Mosadeghi S, Reid MW, Martinez B, Rosen BT, Spiegel BMR. Acceptability of an Immersive Virtual Reality Intervention for Hospitalized Patients With Gastrointestinal Disorders. *Gastro* 2016;150(4,S1):S643.
21. Todd KH, Funk KG, Funk JP, Bonacci R. Clinical significance of reported changes in pain severity. *Ann Emerg Med*. 1996; 27: 485-9.
22. Bird SB, Dickson EW. Clinically significant changes in pain along the visual analog scale. *Ann Emerg Med*. 2001; 38: 639-43.
23. Salaffi F, Stancati A, Silvestri CA, Grassi W. Minimal clinically important changes in chronic musculoskeletal pain intensity measured on a numerical rating scale. *Eur J Pain.* 2004; 8: 283-91.
24. Hjermstad MJ, Fayers PM, Haugen DF, Caraceni A, Hanks GW, Loge JH, Fainsinger R, Aass N, Kaasa S; European Palliative Care Research Collaborative (EPCRC). Studies comparing Numerical Rating Scales, Verbal Rating Scales, and Visual Analogue Scales for assessment of pain intensity in adults: a systematic literature review. J Pain Symptom Manage. 2011 Jun;41(6):1073-93.
25. Cella D, Riley W, Stone A, Rothrock N, Reeve B, Yount S, Amtmann D, Bode R, Buysse D, Choi S, Cook K, Devellis R, DeWalt D, Fries JF, Gershon R, Hahn EA, Lai JS, Pilkonis P, Revicki D, Rose M, Weinfurt K, Hays R; PROMIS Cooperative Group. The Patient-Reported Outcomes Measurement Information System (PROMIS) developed and tested its first wave of adult self-reported health outcome item banks: 2005-2008. J Clin Epidemiol. 2010 Nov;63(11):1179-94.
26. Hays RD, Bjorner JB, Revicki DA, Spritzer KL, Cella D. Development of physical and mental health summary scores from the patient-reported outcomes measurement information system (PROMIS) global items. Qual Life Res. 2009 Sep;18(7):873-80.
27. Üstün TB, Chatterji S, Kostanjsek N, et al. Developing the World Health Organization Disability Assessment Schedule 2.0. Bulletin of the World Health Organization. 2010;88(11):815-823. doi:10.2471/BLT.09.067231.
28. Elliott MN, Lehrman WG, Goldstein EH, Giordano LA, Beckett MK, Cohea CW, Cleary PD. Hospital Survey Shows Improvements in Patient Experience. Health Affairs. 2010;29(11):2061-2067.

HCAHPS Survey

SURVEY INSTRUCTIONS

- You should only fill out this survey if you were the patient during the hospital stay named in the cover letter. Do not fill out this survey if you were not the patient.
- Answer all the questions by checking the box to the left of your answer.
- You are sometimes told to skip over some questions in this survey. When this happens you will see an arrow with a note that tells you what question to answer next, like this:

☐ Yes
☼ No ➔ ***If No, Go to Question 1***

You may notice a number on the survey. This number is used to let us know if you returned your survey so we don't have to send you reminders.
Please note: Questions 1-25 in this survey are part of a national initiative to measure the quality of care in hospitals. OMB #0938-0981

Please answer the questions in this survey about your stay at the hospital named on the cover letter. Do not include any other hospital stays in your answers.

YOUR CARE FROM NURSES

1. During this hospital stay, how often did nurses treat you with courtesy and respect?

1☐ Never
2☐ Sometimes
3☐ Usually
4☐ Always

2. During this hospital stay, how often did nurses listen carefully to you?

1☐ Never
2☐ Sometimes
3☐ Usually
4☐ Always

3. During this hospital stay, how often did nurses explain things in a way you could understand?

1☐ Never
2☐ Sometimes
3☐ Usually
4☐ Always

4. During this hospital stay, after you pressed the call button, how often did you get help as soon as you wanted it?

1☐ Never
2☐ Sometimes
3☐ Usually
4☐ Always
9☐ I never pressed the call button

YOUR CARE FROM DOCTORS

1. **During this hospital stay, how often did doctors treat you with courtesy and respect?**

   1☐ Never
   2☐ Sometimes 3☐ Usually 4☐ Always

2. **During this hospital stay, how often did doctors listen carefully to you?**

   1☐ Never
   2☐ Sometimes 3☐ Usually 4☐ Always

3. **During this hospital stay, how often did doctors explain things in a way you could understand?**

   1☐ Never
   2☐ Sometimes 3☐ Usually 4☐ Always

THE HOSPITAL ENVIRONMENT

4. **During this hospital stay, how often were your room and bathroom kept clean?**

   1☐ Never
   2☐ Sometimes 3☐ Usually 4☐ Always

5. **During this hospital stay, how often was the area around your room quiet at night?**

   1☐ Never
   2☐ Sometimes 3☐ Usually 4☐ Always

CARE Channel Infographic

VR Manual

Samsung Gear VR Instructions

Launching a VR Application

1. Power up phone with the button on the right side of the phone. Hold down until the phone vibrates and turns on.

2. To launch a virtual reality application, select one of icons from the grey dock in the lower screen.
3. Follow the prompt: “To open this application, insert your device into your Gear VR.”

Attach a Mobile Device

1. With the screen facing toward the headset, attach the device to the Gear VR using the **Power/Accessory Interface Connector** (1).
   Note: Make sure the back cover of your mobile device is securely fastened before attaching it to your headset.
2. Press the device into position until the **Phone lock** clicks (2).
3. Attach the window cover to the front of the headset over the mobile device.

Wearing the Gear

1. Align your face and the foam cushioning and put on the Gear **VR. Caution! Do not walk or drive while wearing the Gear VR. Always be aware of your surroundings while using the Gear VR to avoid injury to yourself or others.**

Frequently Asked Questions

How long should a patient wear this?

The experiences run between three and thirty minutes in duration. However, VR experiences are safe as long as the patient is comfortable.

What should be done if a patient does not feel well (dizzy, sick, etc.)?

Please ask the patient to close his or her eyes, which will break the experience, then safely remove the goggles from the patient’s head. Have them sit down for 15 minutes. Make sure they feel better before they leave and check up on them later on that day.

Can I wear my glasses inside of the Samsung Gear VR headset?

No, glasses should not be worn as the product was not designed to accommodate them and your glasses could scratch the non-replaceable lenses inside of the headset. The focus dial located on the top of the headset can be used to adjust the image for nearsighted individuals.

The Gear VR doesn't fit well when I put it on, especially around my nose area. Am I doing something wrong?

There are a number of straps on the headset that can be adjusted for comfort. If you are experiencing discomfort in the nose area, tightening the strap that goes over the top of your head will remove the pressure from the bridge of your nose and move the side straps up from your ears. Tightening too much, however, will shift the weight of the headset on your head. The side straps will adjust the tightness of

the headset and foam on your face. Try not to over-tighten as this will lead to discomfort during longer sessions.

Can I stand up and walk around while using the Samsung Gear VR Innovator Edition?

The Gear VR is designed for seated experiences.

When I plug in the phone after the prompt "to open this application, insert your device into your Gear VR," the application does not load or it loads the wrong screen. Am I doing something wrong?

Please unplug the phone from the Gear VR, return to the home screen with the bottom center button, and restart the process by selecting the application icon and plugging in the phone.

I do not see the application load once I plug in the phone.

Please make sure the Gear VR is completely connected to the phone's Micro USB port.

Samsung's Gear VR Health and Safety Information

Some people (about 1 in 4,000) may have severe dizziness, seizures, epileptic seizures or blackouts triggered by light flashes or patterns, and this may occur while they are watching TV, playing video games or experiencing virtual reality, even if they have never had a seizure or blackout before or have no history of seizures or epilepsy. Such seizures are more common in children and young people under the age of 20. Anyone who has had a seizure, loss of awareness, or other symptom linked to an epileptic condition should consult with a doctor before using the Samsung Gear™ VR Innovator's Edition.

General Instructions and Precautions

- Use only in a safe environment. The Gear VR produces an immersive virtual reality experience that distracts you from and blocks your view of your actual surroundings. Always be aware of your surroundings when using the Gear VR and remain seated at all times. Take special care to ensure that you are not near other people, objects, stairs, balconies, windows, furniture, or other items that you can bump into or knock down when using — or immediately after using— the headset. Do not handle sharp or otherwise dangerous objects while using the Gear VR Innovator's Edition. Never wear the Gear VR in situations that require attention, such as walking, bicycling, or driving.
- Make sure the Gear VR headset is level and secured comfortably on your head, and that you see a single, clear image.
- Ease into using the Gear VR to allow your body to adjust. Use it for only a few minutes at a time at first, and increase the amount of time using the Gear VR gradually as you grow accustomed to virtual reality. Looking around when first entering virtual reality can help you adjust to any small differences between your real world movements and the resulting virtual reality experience.
- A comfortable virtual reality experience requires an unimpaired sense of motion and balance. Do not use the Gear VR when you are tired, need sleep, are under the influence of alcohol or drugs, are hung-over, have digestive problems, are under emotional stress or anxiety, or when

suffering from cold, flu, headaches, migraines, or earaches, as this can increase your susceptibility to adverse symptoms.

- Take at least a 10 to 15 minute break every 30 minutes, even if you don’t think you need it. Each person is different, so take more frequent and longer breaks if you feel discomfort. You should decide what works best.
- Listening to sound at high volumes can cause irreparable damage to your hearing. Background noise, as well as continued exposure to high volume levels, can make sounds seem quieter than they actually are. Due to the immersive nature of virtual reality, do not use the Gear VR with the sound at a high volume, so that you can maintain awareness of your surroundings and reduce the risk of hearing damage.

Discomfort

- Immediately discontinue use if you experience any of the following symptoms while using the Gear VR Innovator's Edition: seizures; loss of awareness; eye strain; eye or muscle twitching; involuntary movements; altered, blurred, or double vision or other visual abnormalities; dizziness; disorientation; impaired balance; impaired hand-eye coordination; excessive sweating; increased salivation; nausea; lightheadedness; discomfort or pain in the head or eyes; drowsiness; fatigue; or any symptoms similar to motion sickness.
- Do not drive, operate machinery, or engage in other visually or physically demanding activities that have potentially serious consequences (i.e., activities in which experiencing any symptoms could lead to death, personal injury, or damage to property), or other activities that require unimpaired balance and hand-eye coordination (such as playing sports, riding a bicycle, etc.) until you have fully recovered from any symptoms.
- Do not use the Gear VR until all symptoms have completely subsided for several hours. Make sure you have properly configured the Gear VR before resuming use.
- Be mindful of the type of content that you were using prior to the onset of any symptoms, because you may be more prone to symptoms based upon the content being used.
- Consult with a doctor if you have serious and/or persistent symptoms.
